# Measles Seroprotection and Catch-Up Vaccination in Medical Students and Other Healthcare Workers at a Large Teaching Hospital: Positive Impact of Mandatory Italian Vaccination Policies

**DOI:** 10.3390/vaccines14090835

**Published:** 2026-09-21

**Authors:** Cristiana Ferrari, Francesco Cama, Lavinia Ponzo, Ilenia Mazzocchi, Claudia Salvi, Lorenzo Ippoliti, Andrea Magrini, Luca Coppeta

**Affiliations:** 1 Department of Biomedicine and Prevention, University of Rome Tor Vergata, 00133 Rome, Italy; lorenzo.ippoliti@unicamillus.org (L.I.); andrea.magrini@uniroma2.it (A.M.); luca.coppeta@uniroma2.it (L.C.); 2 PhD Program in Social, Occupational and Medico-Legal Sciences, Department of Biomedicine and Prevention, University of Rome Tor Vergata, 00133 Rome, Italy; francesco.cama@ptvonline.it (F.C.); lavinia.ponzo@ptvonline.it (L.P.); ilenia.mazzocchi@ptvonline.it (I.M.); claudia.salvi@uniroma2.it (C.S.); 3 Faculty of Medicine, Saint Camillus International University of Health Sciences, 00131 Rome, Italy

**Keywords:** measles, measles IgG, medical students, healthcare workers, occupational health, vaccination, catch-up vaccination, seroprotection

## Abstract

Background: Measles resurgence across Europe has renewed attention to immunity gaps in healthcare environments. Following decades of sub-optimal coverage, Italy implemented mandatory vaccination under Law 119/2017 to counteract declining rates. However, young adults entering medical training represent a transitional cohort with heterogeneous exposures to natural infection versus vaccination. This study evaluated the evolution of measles immunity among medical students and healthcare trainees, assessing the impact of vaccination policies and the operational efficacy of an on-site catch-up vaccination pathway. Methods: We conducted an observational study of 1014 medical-area students and young healthcare workers aged 18–35 years undergoing occupational surveillance in 2025 at Policlinico Tor Vergata, Rome. Measles IgG antibodies were quantified by chemiluminescent immunoassay (VIRCLIA^®^ S/CO > 1.0 defining protection). Findings were compared with baseline institutional data from 2020. Results: Overall seroprotection in 2025 was 91.8% (931/1014). Stratification showed no significant differences by sex (92.6% female vs. 90.1% male; *p* = 0.168), age group (92.8% in 18–25 vs. 90.9% in 26–35 years; *p* = 0.249), or nationality. Among the 83 sub-threshold individuals, 42.2% had documented prior immunization, reflecting secondary humoral waning rather than primary vaccine failure. On-site revaccination raised final seroprotection to 95.4%. Comparative assessment demonstrated a significant progressive shift from natural wild-type exposure toward vaccine-induced immunity over the past decade. Conclusions: Mandatory vaccination policies have stabilized baseline protection, yet waning humoral titers among vaccinated young adults present unique operational challenges. A structured occupational protocol combining serological screening, historical verification, and on-site revaccination effectively closes nosocomial immunity gaps.

## 1. Introduction

Over six decades after the pioneering development of the first attenuated measles vaccines by Nobel Laureate John Enders and colleagues [1], measles remains one of the most contagious vaccine-preventable viral diseases and continues to challenge global elimination programs even in high-income countries [2,3,4]. Its high transmissibility, the possibility of severe complications [5,6,7,8] and the need for population immunity levels close to or above 95% make measles a priority for public health surveillance, particularly in settings where susceptible individuals may encounter fragile patients.

Healthcare facilities are vulnerable to measles transmission because they concentrate exposed workers, trainees, patients with severe diseases and immunocompromised individuals in the same environment [9,10]. For this reason, healthcare workers, residents and medical-area students should have documented evidence of immunity before beginning clinical activities [9,11,12,13,14]. In Italy, occupational health services play a central role in identifying susceptibility and offering vaccination to personnel who lack serological or documentary evidence of protection [11,12,13,14].

The nosocomial transmission dynamics of the measles virus represent a unique and severe challenge within modern healthcare infrastructure. With a basic reproductive number (R_0_) historically estimated between 12 and 18 in susceptible populations, measles stands as one of the most intensely contagious viral pathogens known to medicine [1,15]. The primary mode of transmission relies on airborne droplet nuclei and aerosols generated during coughing or sneezing, which can remain suspended in the air and retain infectious potency for up to two hours in enclosed spaces [9,16]. In a hospital environment, this environmental persistence facilitates cross-infection, transforming waiting areas, emergency rooms, and wards into potential hot spots for outbreaks. The clinical consequences of nosocomial transmission are particularly serious because healthcare facilities concentrate individuals with high physiological vulnerability. Exposure of immunocompromised patients, such as those undergoing active chemotherapy, organ transplant recipients on immunosuppressive regimens, neonates who have not yet reached the age for routine immunization, and pregnant women, may lead to severe complications. These complications include viral pneumonia, acute encephalitis, and subacute sclerosing panencephalitis (SSPE), with substantial morbidity and mortality [1,4,5,6,7,8,9,10,14]. Consequently, healthcare workers (HCWs) and medical-area students, who move across hospital departments during clinical rotations, may contribute to dissemination if they lack presumptive evidence of immunity [9,11,14,16].

The current immune profile of young adults entering medical training in Italy is deeply intertwined with the historical evolution of national vaccination policies over the past three decades. The cohort evaluated in this study—predominantly born between 1990 and 2007—spans a transitional era characterized by shifting public health strategies and varying regional coverage rates. Prior to the systematic implementation of national elimination plans, measles immunization in Italy relied on voluntary adherence, which frequently resulted in heterogeneous coverage across regions and socio-economic strata. In 2003, the Italian Ministry of Health launched the first National Plan for the Elimination of Measles and Congenital Rubella (Piano Nazionale per l’Eliminazione del Morbillo e della Rosolia Congenita—PNEMoRc), which formalized a two-dose schedule using the combined Measles–Mumps–Rubella (MMR) vaccine [17]. Despite these structured guidelines, national coverage periodically remained below the 95% target required for elimination [17,18,19]. This epidemiological vulnerability prompted a major public health response strongly advocated by the Italian Minister of Health Beatrice Lorenzin and leading scientific authorities, including Walter Ricciardi (then President of the Istituto Superiore di Sanità). This culminated in Decree-Law 73/2017, converted into Law 119/2017 (widely known as the ‘Legge Lorenzin’). The legislation mandated ten compulsory childhood immunizations for school registration up to 16 years of age: six mandatory hexavalent components (diphtheria, tetanus, pertussis, hepatitis B, poliomyelitis, and Haemophilus influenzae type b) and four mandatory components against measles, mumps, rubella, and varicella (MMRV) [20]. Although the intervention increased childhood vaccination coverage, it also highlighted a residual cohort of young adults who grew up before the mandate. This group may present incomplete records, undocumented natural infection, or waning antibody titers, underscoring the preventive role of occupational health services in university teaching hospitals [11,12,14,18,19].

Recent European and Italian epidemiological data indicate that measles circulation has increased after the pandemic period, with a relevant proportion of cases occurring in adolescents and adults [21,22,23]. In addition, the estimated prevalence of vaccine-induced measles protection (94.1%) among children in the target vaccination population within the European region was sufficient to prevent the transmission of measles virus strains with R0 ≤ 16, but remained insufficient against strains with higher transmissibility (R0 > 16) [24]. This trend makes the assessment of immunity among young healthcare personnel particularly important. Medical students represent a specific group because many were born during periods of changing vaccination policies [18,19] and may also include international students from countries with different immunization schedules and vaccine-coverage histories.

Measles vaccination coverage in Italy has decreased over the last few decades, creating recurring immunity gaps among adolescents and young adults. In response to dropping coverage and recurrent epidemics, the Italian government enacted Decree-Law 73/2017, converted into Law 119/2017, establishing mandatory immunization for ten childhood vaccines—including measles–mumps–rubella (MMR)—for cohorts born between 2001 and 2017.

Previous institutional studies revealed that a concerning proportion of medical students and healthcare trainees lacked protective antibody levels at the inception of their clinical rotations [25,26,27]. This historical vulnerability reflects the shifting epidemiological landscape: older healthcare workers acquired long-lasting, robust humoral immunity primarily via wild-type exposure, whereas younger cohorts rely almost exclusively on vaccine-induced protection. Vaccine-induced humoral titers are known to decline over decades at a faster rate than post-infection antibodies, despite the persistence of cellular immune memory. Consequently, trainees entering hospital environments may test seronegative or sub-threshold despite having completed recommended childhood immunization schedules.

Building on these prior investigations, the present study aims to evaluate the impact of mandatory vaccination policies on the immunization status of medical-area students and young healthcare workers by comparing current seroprevalence with historical data from 2020. Furthermore, we examine the distinct immunological mechanisms characterizing vaccine-induced waning versus natural immunity, evaluate the clinical significance of sub-threshold IgG titers in fully vaccinated individuals, and assess the operational effectiveness of a hospital-based catch-up protocol in reaching the >95% protective threshold required to prevent nosocomial transmission.

## 2. Materials and Methods

This was a retrospective observational study based on occupational health surveillance data from Policlinico Tor Vergata, Rome. The study population consisted of medical-area students and young healthcare workers aged 18–35 years who underwent medical surveillance at the Occupational Medicine Service between January 2025 and December 2025.

For each participant, demographic information, including age, sex and nationality, was extracted from the available records. Nationality was categorized as Italian or foreign. Foreign participants were additionally described by geographical area of origin when available.

All participants underwent venous blood sampling as part of routine occupational health assessment. Measles-specific IgG antibodies were measured with the MEASLES VIRCLIA^®^ IgG MONOTEST (VCM054; Vircell, S.L., Granada, Spain), an indirect chemiluminescent immunoassay [27,28]. Results were expressed as an antibody index, reported operationally as S/CO in the study dataset. According to the classification used in the updated dataset, IgG values > 1.0 S/CO were considered protective, whereas values < 1.0 S/CO were considered below the protective threshold.

Subjects with acute symptoms suggestive of ongoing infection were not considered eligible for routine serological screening at that time. Available laboratory results were extracted from the Modulab laboratory software.

Participants with measles IgG < 1.0 S/CO were evaluated through review of available vaccination documentation and medical history. When a complete vaccination schedule or natural immunization was documented, subjects were classified as already immunized despite the low serological result. When protection could not be documented, a complete vaccination course or a booster dose with measles-containing vaccine was offered by the Occupational Medicine Service, unless contraindications were present [9,11,13,14,18,19]. Reasons for non-vaccination were categorized as documented immunity, vaccination at Policlinico Tor Vergata, non-response after vaccination, deferral, contraindication or refusal.

Categorical variables were summarized as counts and percentages. Continuous variables were summarized as mean and standard deviation when appropriate. Differences in proportions were assessed using the chi-square test or Fisher’s exact test when appropriate. Statistical significance was defined as *p* < 0.05. Analyses were performed using IBM SPSS Statistics version 25 for Windows. The study was approved by the local Ethics Committee for research involving human subjects.

### 2.1. Detailed Occupational Health Surveillance Workflow

The operational protocol implemented by the Occupational Medicine Service at Policlinico Tor Vergata follows a strictly standardized, multi-step workflow integrated into the mandatory health surveillance program for medical-area students and young healthcare workers. Upon enrollment in their respective clinical tracking systems or immediately prior to hiring, all subjects receive a digital convocation for a preventive medical examination. The workflow proceeds through the following sequential phases:

Initial Clinical Interview and Document Retrieval: A specialized occupational physician conducts a comprehensive anamestic interview focused on past infectious diseases and records any history of clinical measles. Simultaneously, participants are required to submit their official regional vaccination certificates or historical immunization booklets.

Phlebotomy and Serological Screening: Regardless of documented history, all participants undergo venous blood sampling to objectively quantify circulating measles-specific IgG antibodies, ensuring a baseline biological assessment.

Data Integration and Risk Categorization: Laboratory results are paired with the participant’s records. Subjects with protective titers (>1.0 S/CO) are deemed fit for clinical duty without restrictions regarding measles exposure. Subjects presented with sub-threshold titers (<1.0 S/CO) enter the active recall pathway.

Active Recall and Clinical Management: Sub-threshold individuals undergo a secondary, meticulous review of their immunization history. If a verifiable, complete two-dose childhood vaccination schedule is confirmed, they are classified as ‘immunized with low serology’ and cleared. If documentation is incomplete, missing, or absent, they are formally scheduled for the immediate on-site catch-up vaccination pathway [14].

### 2.2. Technical Specifications of the Chemiluminescent Immunoassay (CLIA)

Quantitative determination of serum measles-specific IgG antibodies was performed using the MEASLES VIRCLIA^®^ IgG MONOTEST (VCM054; Vircell, S.L., Granada, Spain), an indirect chemiluminescent immunoassay suitable for automated processing [28]. The method is based on the reaction of antibodies in the patient sample with measles virus antigen adsorbed onto polystyrene reaction wells. Unbound immunoglobulins are removed by washing, after which an enzyme-labelled anti-human IgG conjugate binds to the antigen–antibody complex. Following a second wash, a chemiluminescent substrate containing peroxide and luminol generates glow-type luminescence, which is measured as Relative Light Units (RLU) with a luminometer. The manufacturer defines the antibody index as sample RLU divided by calibrator RLU and classifies values < 0.9 as negative, 0.9–1.1 as equivocal, and >1.1 as positive [28]. To prioritize patient safety in a high-exposure hospital environment with vulnerable and immunocompromised patients, an operational threshold of S/CO > 1.0 was pre-specified to define seroprotection. Consequently, equivocal results in the lower manufacturer equivocal zone (0.9–1.0 S/CO) were categorized as sub-threshold, triggering documentary review and vaccination offer rather than presuming immunity. Baseline comparison with medical students screened in 2020 at the same institution [27] utilized the identical diagnostic platform (MEASLES VIRCLIA^®^ IgG MONOTEST, Vircell) and standardized laboratory workflows at the Policlinico Tor Vergata laboratory, ensuring full methodological consistency between cohorts.

### 2.3. Catch-Up Vaccination Protocol and Clinical Safety Measures

The catch-up immunization intervention used a live attenuated combined Measles–Mumps–Rubella (MMR) vaccine (Priorix or M-M-RVAXPRO) [29,30]. The lyophilized vaccine was reconstituted with the manufacturer-supplied solvent to yield a standard 0.5 mL dose, administered subcutaneously or intramuscularly in the deltoid region by trained occupational health nursing staff. The Occupational Medicine Service maintained the cold chain using medical-grade refrigeration continuously monitored by digital data loggers at +2 °C to +8 °C [29,30]. Before vaccination, subjects underwent safety screening for contraindications and deferral criteria. Contraindications included a history of severe hypersensitivity to relevant vaccine components, pregnancy, and severe primary or acquired immunodeficiency. Moderate or severe acute febrile illness required temporary deferral; recent receipt of immunoglobulins or antibody-containing blood products was assessed according to the product, dose, and recommended interval [14,29,30]. For subjects requiring a complete two-dose primary course, the second dose was scheduled at least 28 days after the first dose [14]. Post-vaccination serological re-evaluation was scheduled 4 to 6 weeks after completion of the assigned course to assess seroconversion.

## 3. Results

The study included 1014 participants aged 18–35 years, with a mean age of 25.91 years (SD 4.065). Women represented 680/1014 subjects (67.1%) and men accounted for 334/1014 (32.9%). Overall, 870 participants (85.8%) were Italian nationals.

Main population characteristics are shown in Table 1.

A detailed descriptive analysis was conducted on the sub-cohort of 144 foreign nationals, representing 14.2% of the total study population, to identify potential variations linked to geographic origin. Most participants originated from Europe (92; 63.9%), primarily Eastern European countries. The remaining participants originated from Asia (32; 22.2%), Africa (9; 6.3%), South America (9; 6.3%), North America (1; 0.7%), and Central America (1; 0.7%). Baseline seroprotection was 93.5% among European participants (86/92) and 90.6% among Asian participants (29/32); all participants from Africa, South America, North America, and Central America were above the study threshold. The 9 foreign participants with sub-threshold IgG levels were distributed across the European (6) and Asian (3) subgroups.

A detailed qualitative evaluation was also performed regarding the clinical reasons for the 8 individuals categorized as ‘vaccination deferred’ and the 2 individuals categorized as ‘vaccination contraindicated’ within the sub-threshold group of 83. Among the deferred cases, 5 were receiving systemic immunosuppressive therapies for dermatological or rheumatological conditions; local clinical criteria required postponement until three months after therapy. The remaining 3 deferrals involved participants undergoing fertility treatment and awaiting confirmatory beta-hCG testing, warranting temporary caution with a live viral vaccine. The 2 contraindications involved confirmed histories of severe hypersensitivity reactions to relevant vaccine components, precluding administration of the live attenuated formulations [29,30].

At baseline, 931 participants (91.8%) had measles IgG values above the protective threshold (>1.0 S/CO), while 83 (8.2%) had IgG values < 1.0 S/CO. (See Table 2).

Seroprotection was slightly higher among women than men, but this difference was not statistically significant (92.6% vs. 90.1%; *p* = 0.168). When the sample was divided into two age groups, subjects aged 18–25 years showed a seroprotection rate of 92.8%, compared with 90.9% among those aged 26–35 years; the difference was not statistically significant (*p* = 0.249).

Seroprotection did not differ significantly according to nationality. Based on the available contingency counts, 796/870 Italian participants (91.5%) and 135/144 foreign participants (93.8%) had IgG values > 1.0 S/CO (chi-square *p* = 0.360 without continuity correction). (See Table 3).

Among the 83 participants with IgG < 1.0 S/CO, 35 (42.2%) were classified as already immunized because a complete vaccination schedule was documented or natural immunization was reported. Thirty-seven subjects (44.6%) were vaccinated at Policlinico Tor Vergata in 2025. One vaccinated subject remained below the serological threshold after completion of the vaccination course and was classified as a non-responder. Among the remaining participants with IgG < 1.0 S/CO, vaccination was deferred in 8 cases (9.6%), contraindicated in 2 cases (2.4%), and refused by 1 subject (1.2%).

Considering the 36 serological responders after on-site vaccination, the proportion of participants with IgG values above the protective threshold increased from 931/1014 (91.8%) at baseline to 967/1014 (95.4%) after the occupational health intervention. (See Table 4).

### Temporal Comparison: 2020 Versus 2025 Cohorts

To address the longitudinal evolution of measles protection within our teaching hospital, we compared the 2025 surveillance findings (*n* = 1014) with baseline data obtained from medical students screened at the same institution in 2020 (*n* = 482). In 2020, baseline seropositivity was 85.7% (413/482), leaving 14.3% of the cohort susceptible. In the 2025 cohort, seroprotection reached 91.8% (931/1014), representing a statistically significant improvement in baseline immunity (Chi-square test = 13.92, *p* < 0.001). Furthermore, while the 2020 cohort exhibited higher rates of undocumented vaccine status, the 2025 cohort demonstrated improved documentation traceability, reflecting the indirect downstream effects of national immunization registry digitization and strengthened pre-matriculation compliance.

## 4. Discussion

This updated analysis shows that most medical-area students and young healthcare workers assessed at Policlinico Tor Vergata had measles IgG levels above the protective threshold. Baseline seroprotection was 91.8%, and the occupational health intervention increased the proportion of serologically protected subjects to 95.4%. This finding is relevant because a threshold close to 95% is generally considered necessary to prevent sustained measles transmission in highly susceptible populations and healthcare environments [1,2,3,9,21,22].

The most important contribution of this updated manuscript is not simply the higher rate of baseline seroprotection compared with earlier work, but the description of the management pathway following screening. Among subjects with IgG below the threshold, a substantial proportion already had documented evidence of vaccination or natural immunization. For those without such evidence, the availability of vaccination within the same occupational health pathway allowed for rapid catch-up immunization. Only one subject refused vaccination, suggesting that logistical accessibility and active counseling may strongly influence acceptance in this population [9,11,12,14,26].

No statistically significant association was found between seroprotection and sex. Women showed a slightly higher protection rate than men, but the difference was small and did not reach statistical significance. This result should be interpreted cautiously because previous studies have reported sex-related differences in immune response to vaccines, while other studies have not confirmed clinically relevant differences in comparable young adult populations [25].

Age was not significantly associated with seroprotection when participants were divided into the 18–25 and 26–35 age groups. The slightly higher seroprotection observed among younger subjects may reflect more recent vaccination policies and a shorter time since immunization, but the present study was not designed to directly evaluate waning immunity because the exact dates of vaccination were not systematically available [31,32].

Nationality was also not significantly associated with baseline seroprotection in this updated cohort. This differs from earlier analyses at the same institution in which Italian students and residents showed higher serological protection than foreign-born trainees [25,26,27]. Several factors may explain this discrepancy, including the different sample size, the lower proportion of foreign participants in the current cohort, the use of a different serological threshold, and the possible effect of previous screening and vaccination activities. Therefore, direct numerical comparison with previous studies should be avoided unless the same assay threshold, population definition and inclusion period are applied.

The presence of subjects with documented vaccination or natural immunization despite IgG values below the threshold highlights a practical challenge for occupational medicine. Serology is useful for identifying potential susceptibility, but antibody levels alone may not fully reflect immune memory or clinical protection after complete vaccination [14,31,32,33]. Nevertheless, in hospital settings, a structured algorithm based on documentation review, serology where appropriate, and vaccination offer remains important for risk management [9,11,14].

The identification of one apparent non-responder is also clinically relevant. Although rare, primary or secondary vaccination failure [15,31,32] raises questions about individualized follow-up, counseling, and decisions during outbreaks. In such cases, the occupational health service should maintain updated documentation and coordinate exposure management with infection-control and public health teams [14,16].

### 4.1. Impact of Mandatory Vaccination Policies and Longitudinal Trends: 2020 vs. 2025

A crucial and novel contribution of this study is the longitudinal assessment of measles immunity across a 5-year interval at the same teaching hospital. Comparing our 2025 findings with institutional baseline data from 2020 [27] reveals a statistically significant increase in baseline seroprotection, rising from 85.7% (413/482) to 91.8% (931/1014; Chi-square test = 13.92, *p* < 0.001).

This upward trajectory reflects a profound generational shift in the incoming student population, driven by the broader systemic impact of Italian mandatory vaccination policies (Law 119/2017). Students screened in 2020 were born predominantly between 1995 and 2001—a period characterized by voluntary immunization, substantial regional heterogeneity, and declining coverage that fell well below the 95% elimination threshold. In contrast, the cohort evaluated in 2025 comprises individuals born up to 2007 who directly or indirectly benefited from the enforcement of Law 119/2017 during their school-age years. Although our single-center observational design cannot formally establish direct causality between Law 119/2017 and increased baseline seroprotection, given the absence of a contemporary unexposed control population in Italy, the 6.1 percentage point improvement coincides chronologically with the entrance of cohorts systematically covered by mandatory immunization.

Beyond seroprevalence rates, this policy shift is mirrored by a marked improvement in documentation traceability. While the 2020 cohort showed frequent gaps in immunization records, the 2025 cohort demonstrated significantly higher availability of verified digital certificates and regional immunization records. Law 119/2017 not only curbed pediatric transmission nationwide, but also spurred the digitization and interoperability of regional vaccine registries across Italy. Consequently, occupational physicians in university teaching hospitals can now rely on verifiable documentary evidence to distinguish between true susceptibility and secondary antibody waning, minimizing unnecessary revaccinations and streamlining pre-clinical clearance.

### 4.2. Immunological Mechanics: Natural Versus Vaccine-Induced Immunity and Antibody Waning

The immunological landscape of current healthcare trainees differs fundamentally from that of previous generations. Natural measles infection induces lifelong, robust humoral titers, maintained by long-lived plasma cells and frequent subclinical boosts during historical periods of endemic viral circulation. In contrast, vaccine-induced immunity relies on live-attenuated strains that elicit lower peak antibody concentrations and exhibit measurable decay over decades in the absence of wild-type boosting [31,32].

Our finding that 42.2% (35/83) of seronegative or sub-threshold individuals possessed verified records of complete childhood vaccination highlights the clinical reality of secondary antibody waning rather than primary vaccine failure. In these individuals, circulating neutralizing IgG may fall below the detection limit of commercial immunoassays, yet anamnestic immune memory often remains intact through measles-specific CD4+ and CD8+ memory T-cells and memory B-cell clones [33]. Upon antigen re-exposure, these cellular compartments mount rapid secondary responses capable of blunting viremia and preventing severe clinical manifestations or onward transmission [15].

While cellular memory typically persists despite declining antibody titers, the theoretical risk of nosocomial transmission in enclosed, high-density hospital departments with immunocompromised patients cannot be entirely excluded. Moreover, while community elimination models establish a 95% herd immunity threshold (R0 = 12–18), the protection level required to completely eliminate nosocomial transmission in high-exposure wards may be even higher. Therefore, maintaining >95% institutional protection serves as a critical baseline, but strict adherence to airborne precautions and personal protective equipment remains indispensable.

From an occupational medicine perspective, interpreting a negative IgG titer in a vaccinated healthcare worker as complete susceptibility is therefore an oversimplification. Current CDC and ACIP guidelines explicitly state that two documented doses of live MMR vaccine constitute presumptive evidence of immunity, regardless of subsequent serological test results [14]. Mandatory screening programs must avoid indiscriminate revaccination protocols that overlook validated vaccination registries, prioritizing booster doses primarily for individuals with undocumented or incomplete schedules.

### 4.3. Targeted Risk Mitigation and Management of the Non-Responder

Post hoc power analysis indicated that our sample (680 females vs. 334 males) provided approximately 41.2% power to detect a 2.5% seroprotection difference at α = 0.05. Similarly, the very small sample sizes within non-European foreign groups provide low statistical power. Thus, the absence of statistically significant differences by sex or nationality should be interpreted with caution, as type II errors cannot be excluded. Regarding the single non-responder, no advanced immunogenetic testing (such as HLA typing) was conducted; HLA-associated non-responsiveness remains a theoretical hypothesis rather than a confirmed etiology in this subject.

Importantly, the identification of a single apparent non-responder (1.2% of the sub-threshold group; 0.1% of the total cohort) who remained below the study threshold after the catch-up course is virtually incapable of eroding institutional herd immunity. When the overwhelming majority of healthcare trainees and clinical staff possess validated immunity, nosocomial transmission chains are effectively disrupted, safely shielding the rare non-responder through comprehensive surrounding herd protection. While variation in the humoral response to measles vaccine has been associated with host factors, including specific human leukocyte antigen (HLA) polymorphisms [34], clinical management should not be based on a single post-vaccination titer alone: two documented, appropriately spaced MMR doses constitute presumptive evidence of immunity even when subsequent measles IgG is negative or equivocal [14]. Occupational health services should maintain retrievable vaccination records and coordinate case-specific decisions with infection-prevention teams and public health authorities. All healthcare personnel entering the room of a patient with suspected or confirmed measles should use recommended respiratory protection and airborne precautions, regardless of presumptive immunity [16]. After an unprotected exposure, healthcare personnel without presumptive evidence of immunity should be excluded from work from the fifth day after the first exposure through the twenty-first day after the last exposure. Post-exposure prophylaxis may consist of MMR vaccine within 72 h for eligible susceptible contacts or immune globulin within 6 days when indicated [14,16].

### 4.4. Policy Implications and Future Research Trajectories

The structural success of the catch-up vaccination pathway described in this study has policy implications for institutional health programs in university teaching hospitals. By establishing a ‘one-stop shop’ model in which serological screening, documentation review, clinical counseling, and vaccine administration are integrated into one service, the hospital reduces the organizational friction that can hinder adult immunization programs [11,12,14,26]. In fragmented pathways, a susceptible student may require referral to an external vaccination center, creating additional opportunities for delay and missed vaccination. The very low refusal rate in this cohort (1.2%) suggests that practical access and personalized counseling during a mandatory health check may be more important than explicit hesitancy in this setting. Future studies should longitudinally follow the 36 serological responders identified after the on-site intervention to characterize the durability of vaccine-induced antibodies in young adulthood [31,32]. Research should also assess whether standardized, scalable assays of measles-specific cellular immunity—including IFN-γ-based or lymphoproliferative approaches—could complement serology in individuals with complete two-dose documentation and low antibody values [33]. Such validation could reduce unnecessary revaccination and improve precision in occupational risk assessment.

### 4.5. Limitation of the Study

This study has several limitations. First, it was an observational, single-center investigation conducted at a single tertiary hospital, and relying on historical data generated by the same research group may introduce single-center systematic bias. Second, precise vaccination dates and complete schedules were not uniformly retrievable across all participants due to historical regional registry fragmentation. Third, seroconversion among catch-up responders was evaluated at 4–6 weeks without multi-year follow-up, precluding conclusions regarding long-term antibody persistence or the timing of future boosters. Fourth, while commercial quantitative IgG CLIA represents the universally accepted and standardized occupational correlate of measles immunity, specialized research assays for cellular immunity were not performed, as they are not required nor routinely indicated in standard occupational clearance pathways. Fifth, post hoc power calculations revealed limited statistical power for sex-stratified comparisons (41.2%) and non-European sub-cohorts, precluding definitive conclusions on subgroup equivalence. Finally, in-depth mechanistic and immunogenetic evaluations were not performed on the single non-responder.

### 4.6. Practical Implications

Measles IgG screening can identify a small but operationally relevant group of susceptible or apparently susceptible medical-area trainees.

A documentation review step is necessary because some subjects with IgG below the serological threshold may have evidence of prior complete vaccination or natural immunization.

On-site vaccination substantially improves post-screening protection and minimizes missed opportunities.

Refusal appears uncommon in this cohort; delays and documentation gaps may be more frequent barriers than explicit vaccine refusal.

A centralized digital vaccination registry would improve occupational health decision-making and reduce unnecessary repeat testing.

*Key Points*

- Baseline measles seroprotection among healthcare trainees rose significantly from 85.7% in 2020 to 91.8% in 2025, aligning with the generational transition following Law 119/2017.
- Over 42% of sub-threshold IgG individuals had documented complete childhood vaccination, highlighting secondary antibody waning rather than primary vaccine failure.
- An integrated “one-stop shop” occupational health pathway achieved 95.4% final institutional protection, with minimal refusal (1.2%).
- Validated two-dose immunization documentation constitutes presumptive immunity, preventing unnecessary revaccination while prioritizing true susceptibles.

## 5. Conclusions

In this updated cohort of medical-area students and young healthcare workers, baseline measles seroprotection was high but still below the level required for optimal control in healthcare settings. The occupational health pathway, combining serological screening, review of documented immunity and immediate catch-up vaccination, increased serological protection to 95.4%. These results support the integration of periodic measles immunity assessment and on-site vaccination into routine occupational health surveillance for healthcare workers and students before clinical exposure [9,11,12,14,18,19,26].

## Figures and Tables

**Table 1:** Demographic characteristics of the study population.

Characteristic	N	%
Total participants	1014	100.0
Female	680	67.1
Male	334	32.9
Age 18–25 years	489	48.2
Age 26–35 years	525	51.8
Italian nationality	870	85.8
Foreign nationality	144	14.2

**Table 2:** Levels of protection for measles in the population.

Serological Category	N	%
IgG > 1.0 S/CO	931	91.8
IgG < 1.0 S/CO	83	8.2
Total	1014	100.0

**Table 3:** Measles seroprotection levels stratified by sex, age group, and nationality.

Variable	Group	IgG > 1.0 S/CO, *n*/N (%)	IgG < 1.0 S/CO, *n*/N (%)	*p* Value
Sex	Female	630/680 (92.6)	50/680 (7.4)	0.168
	Male	301/334 (90.1)	33/334 (9.9)	
Age group	18–25 years	454/489 (92.8)	35/489 (7.2)	0.249
	26–35 years	477/525 (90.9)	48/525 (9.1)	
Nationality	Italian	796/870 (91.5)	74/870 (8.5)	0.360 *
	Foreign	135/144 (93.8)	9/144 (6.2)	

* *p* value calculated using Chi-square test without continuity correction.

**Table 4:** Clinical management and outcomes of participants with measles IgG < 1.0 S/CO.

Management Category Among IgG < 1.0 S/CO Subjects	N	% of IgG < 1.0 Group	% of Total Cohort
Vaccinated at Policlinico Tor Vergata, responder	36	43.4	3.6
Vaccinated at Policlinico Tor Vergata, non-responder	1	1.2	0.1
Documented complete vaccination or natural immunity	35	42.2	3.5
Vaccination deferred	8	9.6	0.8
Vaccination contraindicated	2	2.4	0.2
Vaccination refused	1	1.2	0.1
Total IgG < 1.0 S/CO	83	100.0	8.2

## Data Availability

The datasets analyzed during the current study are not publicly available because they contain occupational health data, but aggregated data are available from the corresponding author on reasonable request, subject to institutional and privacy restrictions.
